# Primary Extrapelvic Umbilical Endometriosis Presenting With Cyclical Umbilical Bleeding: A Case Report

**DOI:** 10.7759/cureus.65473

**Published:** 2024-07-26

**Authors:** Felix Anand Raj, Divya Padmakumar, Pavithra Selvam, Imran Thariq Ajmal

**Affiliations:** 1 Department of General Surgery, Chettinad Hospital and Research Institute, Chettinad Academy of Research and Education, Kelambakkam, IND

**Keywords:** umbilical endometrioma, primary umbilical endometriosis, extra-pelvic endometriosis, cyclical umbilical bleeding, case report, atypical presentation of endometriosis

## Abstract

Primary extrapelvic endometriosis is the presence of endometrial tissue in sites outside the uterine cavity in an individual who has had no prior abdominal surgeries. Various theories have been postulated to describe the etiology of endometriosis. Our case study involves a multiparous patient in her late 40s with no prior abdominal surgeries who presented with bleeding from her umbilicus associated with swelling and pain corresponding to her menstrual cycle. A computed tomography scan of the abdomen detected a homogenous granuloma-like umbilical soft tissue mass. The umbilical nodule and the umbilicus were excised, and the specimen was sent for histopathological examination that validated the diagnosis of an umbilical endometrioma by revealing endometrial glands with stroma involving the dermis. Postoperatively, the patient was symptomatically better and was discharged. Primary umbilical endometriosis can mimic conditions like omphalitis, umbilical granuloma, and umbilical hernia; hence, it is important to understand how to differentiate this case from other diagnoses. This case contextualizes that the likelihood of primary umbilical endometriosis in such unusual presentations must always be considered.

## Introduction

The existence of functioning endometrial tissue outside of the typical uterine cavity is known as endometriosis. About 10% to 15% of the women in their reproductive age and 6% of all perimenopausal women are affected [1,2]. An estimated 0.5% to 1.0% of all patients with extragenital endometriosis have primary umbilical endometriosis, making it a rare condition [3]. The development of spontaneous umbilical endometriosis is thought to result from either the urachus remnants metaplasia or when the endometrial cells from the pelvis are transported through lymphatic and vascular channels [4-6]. Theories like celomic metaplasia and implantation are proposed in the pathogenesis of endometriosis, although a consensus on the most suitable theory is yet to be reached [7,8]. The salient feature of this case was the occurrence of an umbilical endometrioma in a multiparous, perimenopausal patient without a history of abdominal surgery or any other gynecological conditions. Additionally, such a case gives us an insight into how to work on our suspicion of rare possibilities such as primary umbilical endometriosis in the context of an atypical presentation.

## Case presentation

A multiparous perimenopausal patient in her late 40s visited the surgical outpatient department of our institution with complaints of umbilical swelling for six months that was associated with pain and sanguineous discharge from her umbilicus, synchronous with her menstrual cycles. A known case of dyslipidemia on medication for one year, she has not had any prior abdominal surgeries and has had four normal vaginal births, with her most recent one occurring nine years ago.

**Figure 1:**
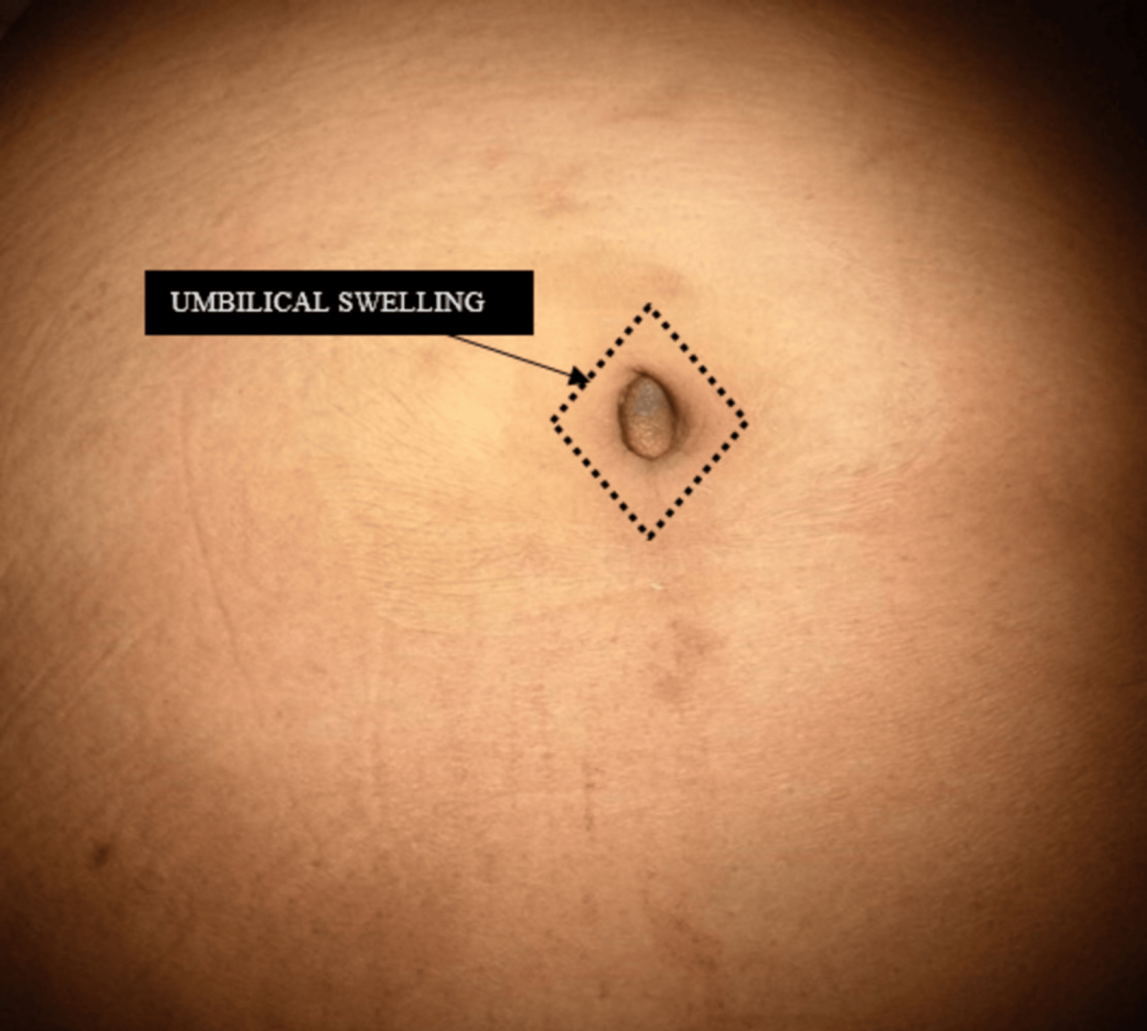
Preoperative image of the livid-colored umbilicus.

Upon general examination, her BMI was 30.5 kg/m^2^, with a decent overall condition and stable vitals. An examination of her abdomen revealed a 3x2 cm umbilical swelling that was firm, nodular, non-tender, and nonreducible, and the skin over the swelling appeared dull grayish (Figure 1). Additionally, there was no cough impulse. Furthermore, the swelling did not exhibit any evidence of inflammation or active discharge during examination. Per rectal examination was normal.

Investigations

**Figure 2:**
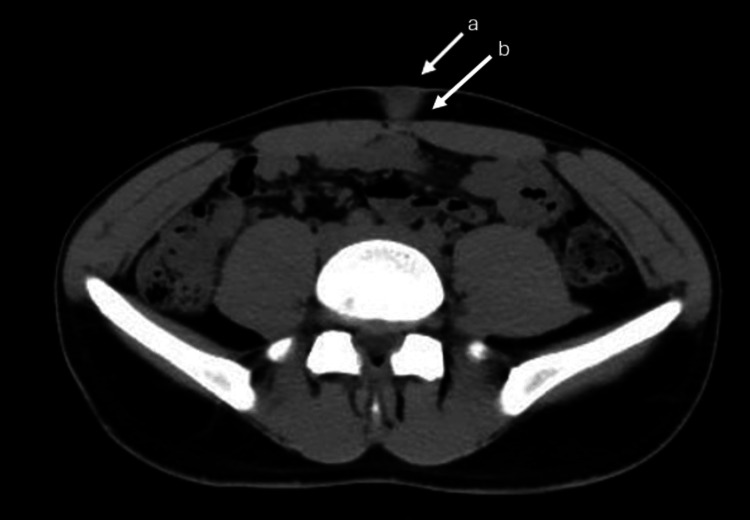
(a) Homogenous granuloma-like umbilical soft tissue mass seen in abdominal computed tomography; (b) thin rim separating the umbilical soft tissue mass from the underlying abdominal muscles.

The patient was duly evaluated with the routine preoperative investigation. On initial ultrasound evaluation, a heterogeneous hypoechoic lesion measuring 2.7x2.2 cm was seen in the umbilicus, and the uterus seemed bulky, suggesting the presence of adenomyosis. Abdominal computed tomography scan revealed a homogenous granuloma-like umbilical soft tissue mass with a thin rim separating the umbilical soft tissue mass from the underlying abdominal muscles (Figure 2).

Treatment and follow-up 

**Figure 3:**
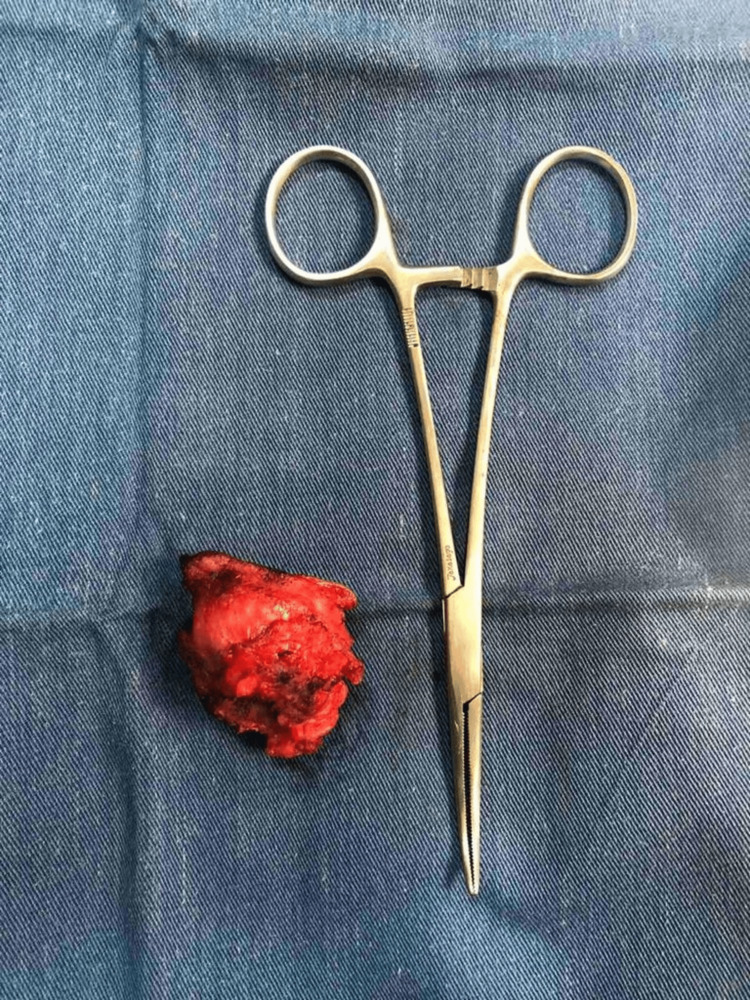
Immediate operative specimen of umbilicus with nodule.

An excision biopsy of the umbilical nodule was planned under spinal anesthesia. An incision was made and deepened, intraoperatively the nodule was well-defined, and densely inseparable from the umbilicus, the nodule was excised completely, and an omphalectomy was performed. The specimen was sent for histopathological examination (Figure 3).

**Figure 4:**
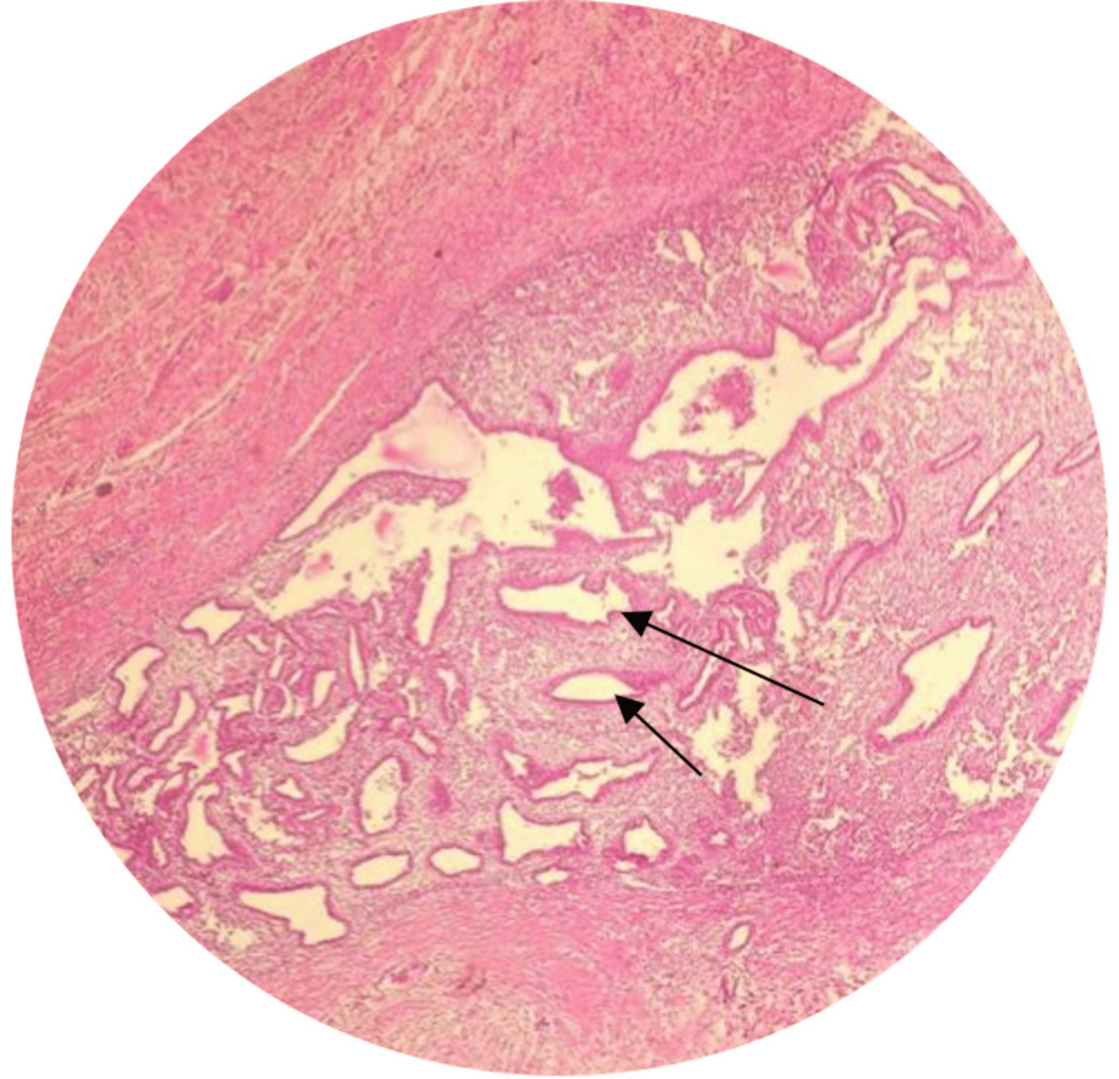
Hematoxylin and eosin staining at 10x magnification shows endometrial glands with stroma involving the dermis and suggestive of endometriosis. Arrows indicate the endometrial glands.

**Figure 5:**
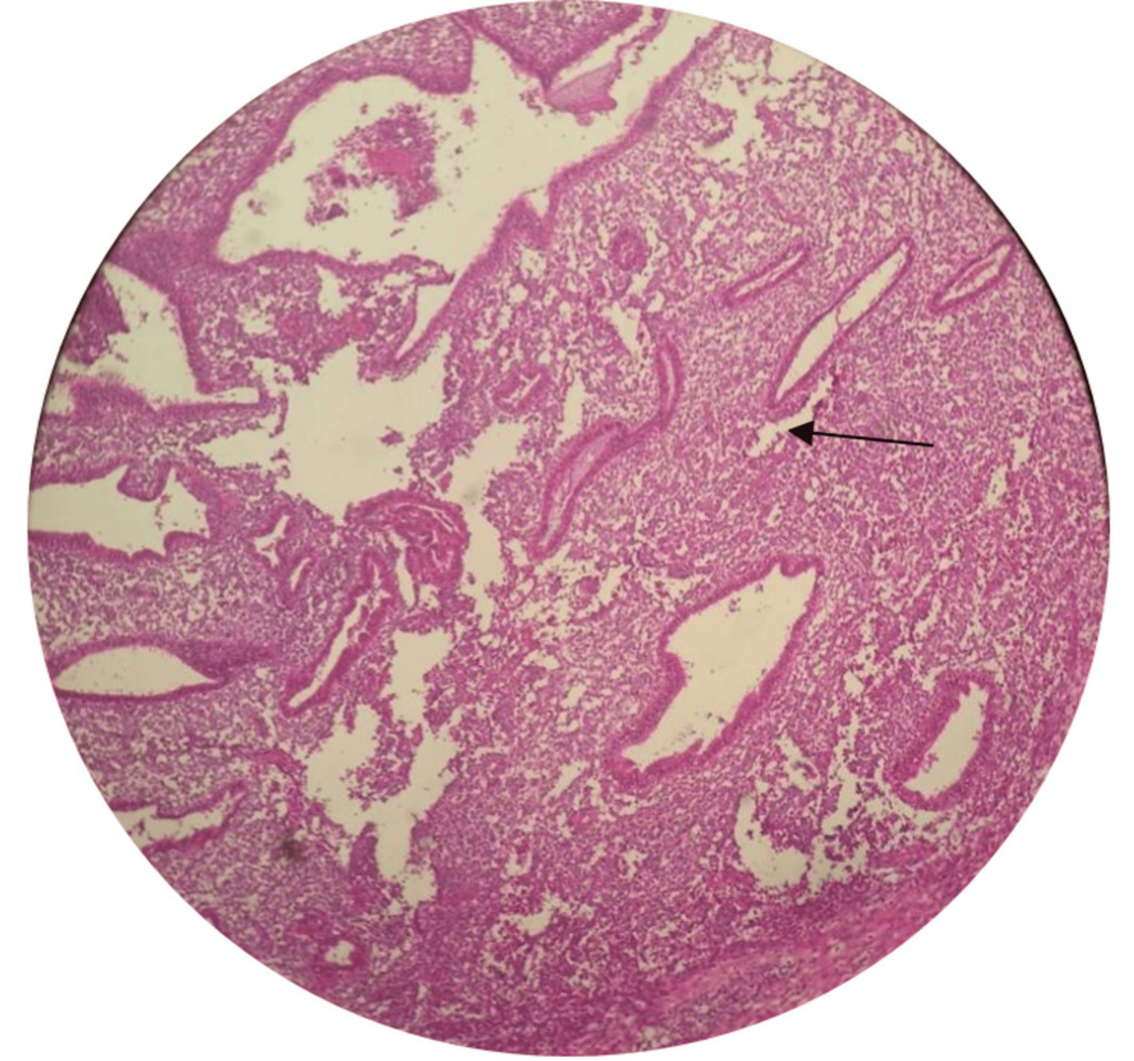
Hematoxylin and eosin staining at 40x magnification showing the endometrial glands with stroma involving dermis. Arrow indicates the endometrial glands.

The cut section of the specimen showed a grayish-white firm area measuring 2x2 cm, and microscopically, the underlying dermis showed islands of endometrial glands and endometrial stroma along with areas of hemorrhage and hemosiderin-laden macrophages and some myxoid changes, suggestive of endometriosis (Figures 4, 5).

The postoperative period was uneventful, and the patient was treated with intravenous antibiotics, analgesics, and other supportive measures. She was discharged on postoperative day five and was able to resume her daily activities with ease. The patient was followed up till six months post-surgery, and all the follow-up visits were uneventful.

## Discussion

The term endometriosis refers to the extrauterine presence of functional endometrial tissue. Up to 15% of women experience it during their lifetime [9]. Even more mysterious than pelvic endometriosis, extrapelvic endometriosis is undoubtedly rarer and more challenging to identify and cure. Endometriosis of the abdominal wall can mimic granuloma, omphalitis, lipoma, sebaceous cyst, umbilical hernia, or primary or metastatic cancer, both clinically and through diagnostic imaging [10-12]. When pelvic and umbilical endometriosis overlap, the notion of hematogenous and lymphatic spread is preferred. In the event of isolated umbilical endometriosis, it is thought that it may develop as a result of metaplasia of the residual urachus (Table 1).

**Table 1:** Various theories describing the etiology of endometriosis

Theories Regarding the Etiology of Endometriosis	Pathophysiology Described by the Theories
Sampson’s Implantation theory (1922)	Retrograde menstruation results in the reflux of endometrial cells via the fallopian tube followed by implantation into the peritoneal cavity and surrounding structures [13]
Meyer and Ivanoff’s Celomic metaplasia theory (1919)	Metaplasia of embryonic cell rests in the embryonic endothelium [13]
Halban’s theory (1924)	Transportation of endometrial fragments through the vascular and lymphatic channels to distant locations [13]
Immune dysregulation	Immune cells such as neutrophils, natural killer cells, macrophages, and T cells spill into the peritoneal cavity causing inflammatory changes that result in endometriosis at pelvic and extrapelvic sites [13]
Hormonal imbalance	Dysregulation between progesterone and estrogen leads to endometriosis [14]
Endometrial stem cell recruitment theory	Migration of stem cells present in menstrual blood to ectopic sites either by retrograde menstruation or during organogenesis of the female reproductive system [15,16]
Micro-RNAs	The presence of an abnormal spectrum of micro-RNA in endometriosis highlights the influence of micro-RNAs in developing endometriosis that can also be considered as a target for medical treatment [17]

Cyclical umbilical pain and bleeding related to menstruation are typically the hallmark clinical signs of umbilical endometriosis [18]. Similarly, in this case, the patient experienced symptoms of a cyclical nature corresponding to the menstrual cycle. Before surgery, the primary purpose of magnetic resonance imaging, ultrasound, and computed tomography is to illustrate the extent of the illness.

Pharmacologic treatment using hormonal drugs, such as progestogens, or surgical excision are the available therapeutic options. However, medical therapy has a poor success record. Thus, the preferred course of treatment is surgical excision and histopathological confirmation of the diagnosis. Both sonographic and computed tomography findings were nonspecific in our case. Hence, the patient was planned for surgical excision following which the specimen was sent for histopathological examination for a confirmatory diagnosis.

## Conclusions

Endometriosis affecting the anterior abdominal wall as a result of cesarean section and sterilization scars is frequently seen in surgical and gynecological outpatient departments. It is quite uncommon for the extrapelvic area, especially the umbilicus, to be affected without any prior surgical scarring. Cyclical umbilical discharge, especially sanguineous, should raise the suspicion of umbilical endometriosis. Umbilical endometriosis is often presented as an umbilical mass of variable size and localized symptoms like cyclic pain and bleeding and has to be differentiated from other common conditions such as omphalitis, granuloma, and umbilical hernia. A complete excision with subsequent histopathological examination is advised for a definitive diagnosis and the best course of treatment.
